# Psychometric properties of the Brazilian version of the Patient Satisfaction Questionnaire

**DOI:** 10.1590/0103-644020256206

**Published:** 2025-04-14

**Authors:** Luisa Gatti-Reis, Renata Negreiros Alvarenga, Xiangqun Ju, Lisa Jamieson, Lucas Guimarães Abreu, Saul Martins Paiva

**Affiliations:** 1 Department of Paediatric Dentistry, Universidade Federal de Minas Gerais (UFMG), Belo Horizonte, MG, Brazil.; 2 Australian Research Centre for Population Oral Health, Adelaide Dental School, University of Adelaide, Adelaide, SA, Australia.

**Keywords:** evidence-based dentistry, validation study, patient-centered care, patient satisfaction, malocclusion

## Abstract

This study aimed to assess the psychometric properties of the Brazilian version of the Patient Satisfaction Questionnaire (PSQ) which assesses the satisfaction of adolescents with orthodontic treatment. The PSQ is a 58-item self-report instrument presenting six subscales (doctor-patient relationship, situational aspects, dentofacial improvement, psychosocial improvement, dental function, and a residual category). The psychometric properties of the Brazilian version of the PSQ (B-PSQ) were assessed in a cross-sectional study with 111 adolescents between 11-18 years who had undergone orthodontic treatment. The sample's features and the questionnaire's characteristics (including the determination of the floor and ceiling effect) were assessed with descriptive statistics. The instrument's internal consistency was assessed by Cronbach’s alpha and stability with the intraclass correlation coefficient (ICC) in test-retest reliability. The convergent and discriminant construct validity was also assessed. Data included responses from 106 adolescents, of which 58 (54.7%) were female. The percentage of individuals reaching the floor effect was 0% in all subscales. In four subscales, the percentage of individuals reaching the ceiling effect was <15%. The B-PSQ showed excellent reliability for the total score (Cronbach’s alpha=0.919; ICC=0.918), ranging from good to excellent for all subscales. B-PSQ’s total score exhibited a high correlation with all five subscales (ρ>0.50). Scores of young adolescents (10-14 years) were significantly higher in the dental function (*p*=0.018) and situational aspects (*p*=0.003) subscales, showing the discriminant validity of the instrument. The B-PSQ has good psychometric properties regarding stability and appears to be a valuable instrument to assess the satisfaction of Brazilian adolescents with orthodontic treatment.

## Resumo

Este estudo teve como objetivo avaliar as propriedades psicométricas da versão brasileira do *Patient Satisfaction Questionnaire* (PSQ), que avalia a satisfação de adolescentes com o tratamento ortodôntico. O PSQ é um instrumento de autorrelato de 58 itens que apresenta seis subescalas (relação médico-paciente, aspectos situacionais, melhora dentofacial, melhora psicossocial, função dentária e uma categoria residual). As propriedades psicométricas da versão brasileira do PSQ (B-PSQ) foram avaliadas em um estudo transversal com 111 adolescentes entre 11 e 18 anos submetidos a tratamento ortodôntico. As características da amostra e as características do instrumento (incluindo a determinação do *floor* e *ceiling effect*) foram avaliadas com estatística descritiva. A consistência interna do instrumento foi avaliada pelo alfa de Cronbach e a estabilidade com o coeficiente de correlação intraclasse (CCI) em um teste-reteste. A validade de construto convergente e discriminante também foram avaliadas. Os dados incluíram respostas de 106 adolescentes, dos quais 58 (54,7%) eram do sexo feminino. A porcentagem de indivíduos que atingiram o *floor effect* foi de 0% em todas as subescalas. Em quatro subescalas, a porcentagem de indivíduos que atingiram o *ceiling effect* foi <15%. O B-PSQ apresentou excelente confiabilidade para o escore total (alfa de Cronbach=0,919; ICC=0,918), variando de bom a excelente para todas as subescalas. O escore total do B-PSQ apresentou alta correlação com todas as cinco subescalas (ρ>0,50). Os escores de adolescentes jovens (10-14 anos) foram significativamente maiores nas subescalas função dentária (p=0,018) e aspectos situacionais (p=0,003), mostrando a validade discriminante do instrumento. A versão brasileira do Patient Satisfaction Questionnaire demonstrou propriedades psicométricas satisfatórias em relação à estabilidade/confiabilidade e parece ser um instrumento útil para avaliar a satisfação dos adolescentes brasileiros com o tratamento ortodôntico

**Figure:**
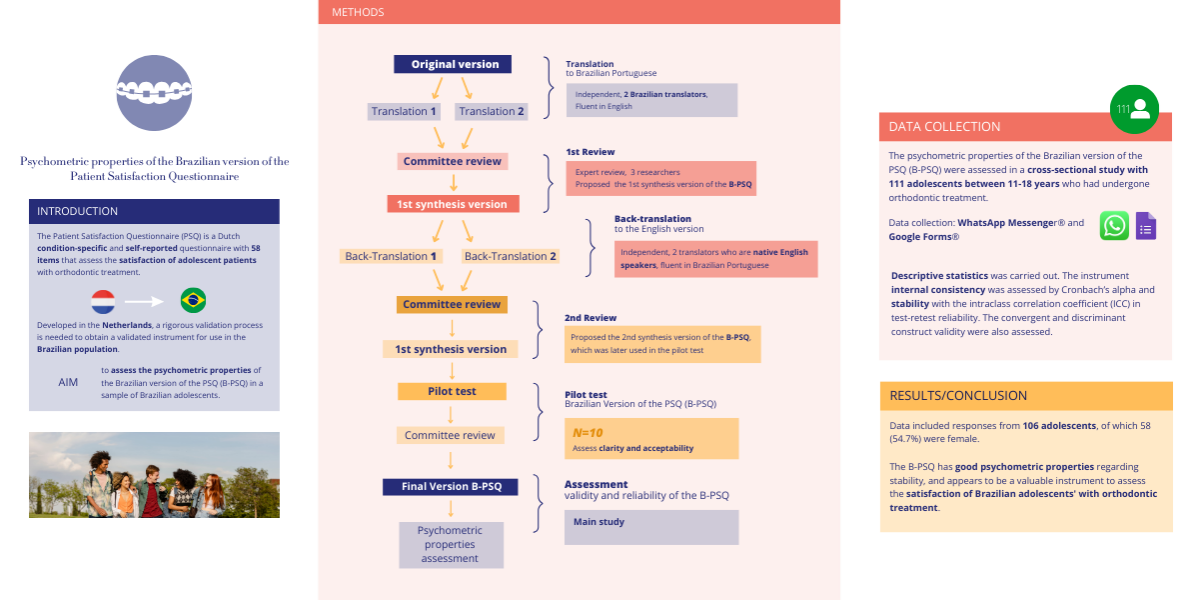

## Introduction

The American Dental Association defines evidence-based dentistry as the result of integrating science, clinicians’ experience, and patients’ voices to make clinical decisions ^1^. Randomized clinical trials (RCTs) of high quality provide valuable information for evidence-based orthodontic practice ^2^. One way to advance evidence-based orthodontics is incorporating patient perspectives as outcomes in clinical trials using Patient Reported Outcome Measures ^2^. The use of a PROM in a population different than the original warrants previous cross-cultural adaptation. In this way, the development and validation of instruments are relevant for innovative orthodontic research and have been the focus of recent studies ^3^ ^,^ ^4^.

In health services research, patient satisfaction has historical roots, tracing back to social movements, concepts used in marketing, and the characteristics of the healthcare system itself ^5^. Satisfaction with health services has been defined as a subjective, dynamic, and multidimensional construct ^5^. In Orthodontics, patient satisfaction holds significant importance, as it is directly associated with the improvement of healthcare services, with services of higher quality, treatment tailored to the needs of the individual, and increased patient adherence ^6^. In 2001, Bennett et al. ^(^ ^7^ designed a questionnaire to assess the satisfaction of parents/caregivers whose children/adolescents had undergone orthodontic treatment. The Brazilian version of this questionnaire has demonstrated excellent psychometric properties ^3^. Nevertheless, during the use of a proxy instrument, there is the likelihood of introducing a proxy respondent bias ^8^. To reduce the possibility of this bias, it is highly recommended that questions related to behaviors, opinions, and beliefs should be avoided in proxy measures ^8^. In addition, given the very complex nature of the outcome satisfaction with health services, relying solely on parents’/caregivers’ reports to evaluate satisfaction with orthodontic treatment may produce limited results due to the low level of agreement between self and proxy reports ^9^.

The Patient Satisfaction Questionnaire (PSQ) is a Dutch condition-specific and self-reported questionnaire with 58 items that assess the satisfaction of adolescent patients with orthodontic treatment ^10^. The PSQ was adapted from a 38-item instrument that had been developed in the United States to assess the satisfaction of ortho-surgical patients ^11^. The PSQ has proved to be a reliable and valid instrument ^10^ and has since then been used in several countries ^4^ ^,^ ^12^. However, as it was developed in the Netherlands, a previous step consisting of cross-cultural adaptation and validation is needed to obtain a validated instrument for use in a Brazilian population. The Brazilian version (B-PSQ) has already been tested concerning the conceptual, semantic, item, operational, and measurement equivalence ^12^. However, the evaluation of the functional equivalence of the instrument, that is, the assessment of how well it performs in terms of psychometric properties has yet to be carried out ^13^. Therefore, the study aimed to assess the psychometric properties of the B-PSQ in a sample of Brazilian adolescents.

## Materials and methods

### Study design

The Institutional Review Board of the Universidade Federal de Minas Gerais approved the protocol of this study (06898519.4.0000.5149) on May 7^th^, 2019. All methods were carried out according to institutional protocols. The reporting of this study was carried out according to the COSMIN reporting guideline ^14^, see Appendix 1. This was a cross-sectional study.

The PSQ is a condition-specific, self-report instrument designed to assess the satisfaction of adolescent patients with orthodontic treatment. It is comprised of 58 items distributed across six subscales (Appendix 2): doctor-patient relationship, situational aspects, dentofacial improvement, psychosocial improvement, dental function, and a residual category. The answer to each item is based on a six-point Likert scale, ranging from endpoints 1 to 6 (1=completely disagree; 2=disagree; 3=slightly disagree; 4=slightly agree; 5=agree; 6= completely agree). Eleven items have a negative connotation and the score of the response of these items should be reversed. The total score of the questionnaire is obtained by adding up the score of the six subscales. The higher the total score, the higher the satisfaction of the respondent. Scores for the six subscales are also possible ^10^.

**Figure 1:**
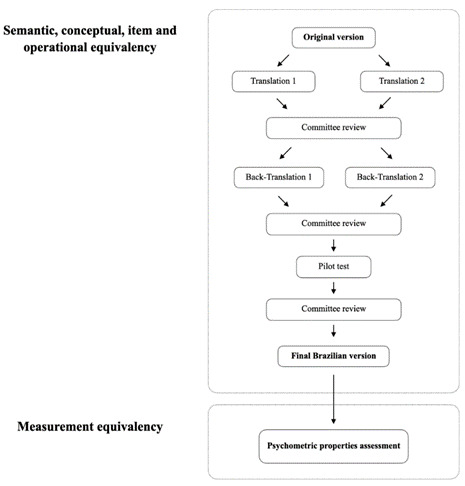
Flowchart presenting the methods used to validate the Brazilian version of the Patient Satisfaction Questionnaire (B-PSQ) in a sample of adolescents

The translation and cross-cultural adaptation of the PSQ to obtain a version in Brazilian Portuguese (B-PSQ) with semantic equivalency followed a Universalist approach. The methods have been previously published ^12^. In this study, the B-PSQ was answered based on a five-point Likert scale with a mid-point. Figure 1 displays the pathway of the complete method used to obtain the semantic equivalency until the measurement equivalency, which is the aim of this study.

### Participants

Adolescent patients who had completed orthodontic treatment with fixed metallic appliances at four orthodontic clinics were invited to participate: three of them were located in Juiz de Fora, Minas Gerais, Brazil and one was located in Leopoldina, Minas Gerais, Brazil. In addition, adolescents who had undergone treatment at a Graduate Program in Orthodontics at *Associação Brasileira de Odontologia,* Juiz de Fora, Brazil, were also invited to participate. Regarding eligibility criteria, adolescents who were native speakers of Brazilian Portuguese, aged 11-18 years, and had completed orthodontic treatment within 0-36 months were included. The time since orthodontic appliance removal was categorized as follows: 0-12 months; 13-24 months; and 25-36 months. Patients who had undergone orthognathic surgery, or had previous diagnosis of craniofacial/cognitive disability reported by parents/caregivers were excluded.

### PROM administration

Orthodontists provided contact information from eligible individuals. Data collection was carried out in two phases. At first, one researcher consulted the patient’s records and extracted the following data: date of birth, Angle’s malocclusion before treatment, and appliance bonding/debonding dates. Angle’s malocclusion before treatment was assessed through the evaluation of pre-treatment photographs. In the next phase, data collection was carried out online, using two platforms: WhatsApp Messenger^®^ and Google Forms^®^. Data collection was carried out remotely given that semantic equivalency of the B-PSQ had been reached using the same methods.

### Data collection procedures

Data collection was carried out from October/2021 to September/2022. Parents/caregivers of eligible individuals were contacted by phone or via WhatsApp Messenger^®^ to inquire whether we could get in touch with their children/adolescents to invite them to volunteer. Each adolescent was contacted using WhatsApp Messenger^®^. As a strategy to maximize volunteer participation, we sent a short one-minute informative video in which one researcher explained the objective of the study and highlighted the importance of their contribution. The video was recorded using the camera of a MacBook Air M1 (Apple Inc., Cupertino, California) and edited using the software Canva (https://www.canva.com). Volunteers received a Google Forms^®^ link containing the two instruments used for data collection: the B-PSQ and a sociodemographic questionnaire. Sociodemographic variables included participants’ sex, self-reported skin color according to the criteria provided by the *Instituto Brasileiro de Geografia e Estatística* (black, brown, indigenous, white, yellow), parent/ caregivers’ sex, schooling, and family income. To assess family income, the Brazilian Monthly Minimum Wage (BMMW) was used, which was approximately USD 220.00 at the time of data collection. To volunteer, adolescents aged below 18 years filled out an assent form, and their parents/caregivers electronically signed a consent form. To assess instrument stability, after three weeks, we sent another Google Forms^®^ link using which participants answered the B-PSQ again.

### Power/sample size calculation

Sample size calculation varies across validation studies ^15^. In this study the recommendation of Anthoine et al. ^15^ was followed: a ratio of person per instrument item ranging from 1.2 to 10; we have chosen to include in the sample 2 individuals per item.

### Statistical analysis

Data analyses were carried out using the software Statistical Package for the Social Sciences (SPSS for Mac, version 25.0; IBM Corp., Armonk, N.Y, USA). Descriptive analysis of sociodemographic data and the B-PSQ were carried out. Missing data will be handled accordingly. Ideally, the expected frequencies of respondents who score the highest or lowest score in an instrument should be below 15%, those who score more are identified as ceiling (highest) and floor effect (lowest) ^16^.

The internal consistency of the questionnaire was assessed using Cronbach's α Coefficient; values of 0.70 or higher were considered acceptable ^17^. Instrument stability was evaluated through test-retest using the Intraclass Correlation Coefficient (ICC). The ICC values were considered weak (ICC < 0.20), fair (0.20 ≤ ICC ≤ 0.40), moderate (0.41 ≤ ICC ≤ 0.60), good (0.61 ≤ ICC ≤ 0.80), and excellent (0.81 ≤ ICC ≤ 1.00) ^18^.

Construct discriminant validity was assessed by comparing the score of each subscale and the total score of adolescents from two age groups. According to the WHO, individuals aged from 10 to 14 years are considered young adolescents ^19^, and individuals aged from 15 to 24 as youth ^20^. The distribution of the total score of the B-PSQ and the score of each subscale was assessed using the Kolmogorov-Smirnov test (N > 50), and normal distribution was not confirmed. Hence, in this analysis, the Mann-Whitney test was used, and the significance level was set at 5%.

The construct convergent validity was assessed using Spearman’s Correlation test between the total score of the questionnaire and the score of each subscale; a significance level of 5% was considered. Spearman’s ρ was considered as small (0.00 ≤ ρ < 0.30), medium (0.31 ≤ ρ ≤ 0.50), and large (ρ > 0.50).

## Results

Of the 111 adolescents recruited to participate in this study, 106 (95.40%) volunteered. The response rate for the analysis of test-retest reliability was 81.30%. There were no missing data. Participants were predominantly female (54.70%) with a mean age of 15.6 ± 1.7 years (Table 1). The majority of adolescents exhibited Angle’s Class I malocclusion (41.50%), had completed their treatment in less than 12 months (39.60%), and were white individuals (78.30%).

**Table 1:** Sociodemographic characteristics of the sample, malocclusion, and time since appliance removal.

	Frequency (%)
Adolescent variables	
Sex	
Male	48 (45.3)
Female	58 (54.7)
Age (years)	
11 years	1 (0.9)
12 years	4 (3.8)
13 years	9 (8.5)
14 years	12 (11.3)
15 years	23 (21.7)
16 years	22 (20.8)
17 years	18 (17.0)
18 years	17 (16.0)
Malocclusion	
Class I	44 (41.5)
Class II Division I	35 (33.0)
Class II Division II	12 (11.3)
Class III	15 (14.2)
Time since appliance removal**	
0 ≤ 12	42 (39.6)
13 ≤ 24	37 (34.9)
< 25 ≤ 36	28 (26.4)
Self-declared race (IBGE criterion)	
Black	6 (5.7)
Brown	16 (15.1)
Indigenous	0 (0.0)
White	83 (78.3)
Yellow	1 (0.9)
Parents/Caregivers’ variables	
Sex	
Male	26 (24.5)
Female	80 (75.5)
Level of Schooling	
≤ 12 years	58 (54.7)
> 12 years	48 (45.3)
Family income (BMW/month)*	
< 3	34 (32.1)
3 ≤ 9	44 (41.5)
< 9	28 (26.4)

The descriptive analysis and the measurements of reliability of the B-PSQ are shown in Table 2. In two subscales (doctor-patient relationship and dental function), the percentage of individuals reaching the maximum score (ceiling effect) was higher than 15%. In all subscales and the total score of the B-PSQ, the percentage of individuals reaching the minimum score (floor effect) was 0%. Regarding the internal consistency analysis, the Cronbach’s α coefficient of the total score was 0.919. For the subscales, Cronbach's α ranged from 0.704 (dentofacial improvement) to 0.906 (psychosocial improvement).

**Table 2:** Descriptive analysis and reliability of the Brazilian version of the Patient Satisfaction Questionnaire

	Number of items	Score range	Mean (SD)	Median (P25-P75)	Floor effect %	Ceiling effect %	Cronbach α	ICC
Doctor-patient relationship	11	11-55	52.92 (2.66)	54.00 (52.00-55.00)	0	35.8	0.791	0.791
Situational aspects	15	15-75	62.89 (5.84)	64.00 (59.75-67.00)	0	0	0.864	0.865
Dentofacial improvement	9	9-45	38.35 (5.18)	40.00 (35.00-41.00)	0	5.7	0.704	0.706
Psychosocial improvement	9	9-45	31.66 (8.48)	32.50 (26.00-39.00)	0	4.7	0.906	0.905
Dental function	4	4-20	16.90 (3.41)	18.00 (14.00-20.00)	0	37.7	0.763	0.751
Residual category	10	10-50	41.25 (4.48)	42.00 (38.00-45.00)	0	0.9	0.767	0.767
Total score	58	58-290	243.97 (19.18)	249.00 (229.50-258.00)	0	0	0.919	0.918

SD=standard deviation, ICC=intra-class correlation coefficient.

The analysis of discriminant validity analysis showed that younger adolescents (10-14 years) exhibited significantly higher scores than older adolescents (15-18 years) for the situational aspects (*p*=0.003) and dental function (*p*=0.018) subscales (Table 3). Convergent construct validity showed a high correlation (ρ>0.50) between the total score and the score of five subscales, in addition to a high correlation (ρ>0.50) between the subscale's doctor-patient relationship and situational aspects (Table 4).

**Table 3:** Discriminant validity, comparison between adolescents of different age groups*

	Age (years)				
	YA Mean (SD)	Y Mean (SD)	YA Median (P25-P75)	Y Median (P25-P75)	p-value**
Doctor-patient relationship	53.69 (1.61)	52.68 (2.88)	54.00 (53.00-55.00)	53.00 (52.00-55.00)	0.111
Situational aspects	65.77 (4.43)	61.95 (5.96)	66.00 (63.00-69.00)	63.00 (58.00-66.00)	0.003
Dentofacial improvement	38.19 (6.41)	38.40 (4.76)	41.00 (37.00-41.00)	40.00 (35.00-41.75)	0.787
Psychosocial improvement	31.42 (9.33)	31.74 (8.24)	31.00 (25.50-41.25)	33.00 (26.25-38.75)	0.869
Dental function	17.96 (3.41)	16.55 (3.36)	20.00 (16.00-20.00)	17.00 (14.00-20.00)	0.018
Residual category	41.58 (4.26)	41.15 (4.58)	43.00 (39.21-44.25)	42.00 (38.00-45.00)	0.672
Total score	248.62 (18.92)	242.46 (19.14)	254.50 (240.00-259.00)	247.00 (226.25-257.75)	0.104

SD=standard deviation, *WHO: Young Adolescents (YA) 10-14 years; Youth (Y): 15 ≤ x≤ 24 years **Mann-Whitney, significant at p<0.05

**Table 4:** Convergent construct validity, Spearman's correlation.

	Doctor-patient relationship	Situational aspects	Dentofacial improvement	Psychosocial improvement	Dental function	Residual category	Total score
Doctor-patient relationship	1	0.533*	0,007	0.108	0.355*	0.345*	0.485*
Situational aspects		1	0.142	0.257*	0.353*	0.316*	0.663*
Dentofacial improvement			1	0.478*	0.391*	0.183	0.586*
Psychosocial improvement				1	0.437*	0.149	0.736*
Dental function					1	0.286*	0.667*
Residual category						1	0.536*
Total score							1

*p<0.01

## Discussion

In the past three decades, RCTs in orthodontic literature were authored by researchers affiliated with different countries and institutions all over the world ^2^. Recent bibliometric data highlighted the role of Brazil: it was the third most productive country, with 94 publications (8.4%). In addition, the University of São Paulo was the most productive institution worldwide ^2^. It is noteworthy the potential the country has to advance the knowledge base in orthodontic clinical trials, which reinforces the need for validated PROMs to be used in data collection. A recent consensus aimed to establish recommendations for outcomes that should be used in clinical trials involving orthodontic patients (except orthognathic and cleft individuals) ^21^. The authors highlighted 7 core outcome sets that were distributed in 4 domains. The patient satisfaction instrument validated in this study enables assessment of the patient's voice regarding both the "clinical" and the "perceived health status" domains.

In healthcare, variables of interest are frequently abstract constructs that require appropriate instruments for a reliable assessment. A matter of great concern is the reliability and validity of instruments ^22^. Cronbach’s coefficient is largely used to verify how well items of the subscale and the items that make up the entire questionnaire correlate ^22^. In this study, the B-PSQ demonstrated coefficients above 0.70 for each subscale and 0.919 for the total score, demonstrating satisfactory internal consistency. In the original study ^10^, the PSQ also performed well, with a coefficient of 0.87 for the total score and 0.70 or higher for each subscale equal to 0.70. In the validation study in England, the authors validated the questionnaire without subscales, as the British version had only one scale with 37 items with a Cronbach's coefficient of 0.92 ^4^.

As for instrument stability, ICC values for the subscales varied from good (0.706) to excellent (0.905) and were also excellent for the total score (0.918). The original study of the PSQ did not assess instrument stability ^10^. In the validation of the British version, Cohen's Kappa Coefficient was used to assess instrument stability, and a value of 0.39 (poor) was observed ^4^. The difference in the level of stability reported herein and in the British study is noticeable. One possible explanation might be the rate of answers for the test-retest: in the validation of the Brazilian version, an 81.3% answer rate for the retest was obtained; in the validation of the British version, though, a much lower rate of 16.5% was found. In addition, the British version had fewer items (that make up a questionnaire without subscales) than the original PSQ or the Brazilian version. In the validation of the Brazilian version, the percentage of participants who reached the lowest score (floor effect) was 0% in all subscales and the total score. The absence of floor/ceiling effect has been highlighted as a measure of quality criteria in health instruments ^16^.

To validate an instrument, the measures aiming to assess construct validity (convergent validity and discriminant validity) are highly encouraged ^23^. In the analysis of convergent construct validity, it is expected that measures that assess similar underlying constructs would correlate positively ^21^ ^,^ ^22^. Similar to what was found in the development of the original questionnaire, the total score of the B-PSQ exhibited a high correlation (ρ>0.50) with four subscales: situational aspects, dentofacial improvement, psychosocial improvement, and dental function.

In the assessment of discriminant construct validity, it was expected a difference in the construct of patient satisfaction between two categories of a variable that is different ^23^. However, adolescence is a period that encompasses great changes in cognitive development, hormonal changes, and ambitions ^19^. The motivations for orthodontic treatment may vary among adolescent individuals from different age groups. In this way, discriminant validity was assessed by comparing the scores of the subscales and the total score of the instrument between young adolescents (from 11-14 years) and youth (from 15 to 18 years) ^19^. In this study, young adolescents exhibited significantly higher scores for the situational aspects and dental function dimensions in comparison to the scores of individuals in the youth age group, indicating greater satisfaction of the young adolescents with respect to both subscales.

The available evidence is conflicting in this regard. A systematic review concluded that patient’s sex and age were not factors associated with patient satisfaction after orthodontic treatment ^24^. More recent studies, however, have been carried out and different results have been obtained. In one study, higher patient satisfaction scores were observed among older adults ^25^. In another study using a non-validated questionnaire, no differences between sexes and age groups and treatment satisfaction were observed ^26^. More evidence to better understand the association between patient satisfaction and age among adolescent patients is needed.

The validation of the B-PSQ has strengths that must be highlighted. The first refers to the moment when participants answered the questionnaire ^10^. During the development of the original instrument, the authors stated that all participants had filled out the questionnaire 3 years after treatment ^10^. In the validation of the British study, participants answered the questionnaire at different time points; however, the time since debonding had not been recorded for 42.7% of respondents ^4^. In this study, adolescents answered the B-PSQ in different intervals (0-12 months; 13-24 months; 25-36 months) and most participants provided their answers within the first year after appliance debonding. In addition, data collection was carried out during difficult times of the COVID-19 pandemic, when Brazil was facing high levels of infection ^27^. The innovative method used herein with satisfactory results shows new possibilities for validation studies using the internet for data collection.

This study also has some limitations. Future studies should focus on the use of the B-PSQ in a large and representative sample of Brazilian adolescents. In addition, the B-PSQ is a long-form instrument that assesses patient satisfaction according to a multidimensional framework. The use of a long-form questionnaire, however, might result in bias, such as response fatigue bias ^8^. In this way, future studies should work on the development of a shorter version of the questionnaire, widening the possibilities for its use.

## Conclusion

The Brazilian version of the Patient Satisfaction Questionnaire has demonstrated satisfactory psychometric properties regarding stability/reliability and appears to be a valuable instrument for assessing the satisfaction of Brazilian adolescents with orthodontic treatment.
